# The single-cell atlas of short-chain fatty acid receptors in human and mice hearts

**DOI:** 10.3389/fimmu.2025.1538384

**Published:** 2025-04-16

**Authors:** Xiaojun He, Qiang Wang, Qiang Long, Yiming Zhong, Zhaoxi Qi, Yecen Zhang, Lan Chang, Bei Qian, Shixing Huang, Xinming Wang, Xuemei Chen, Feifei Li, Xiaomei Yang, Wei Dong Gao, Zhizhao Song, Li Xu, Qiang Zhao

**Affiliations:** ^1^ Cardiovascular Medical Center, Department of Cardiovascular Surgery, Nanjing Drum Tower Hospital, Affiliated Hospital of Medical School, Nanjing University, Nanjing, China; ^2^ Department of Cardiovascular Surgery, Ruijin Hospital, Shanghai Jiaotong University School of Medicine, Shanghai, China; ^3^ Department of Anesthesiology, Shanghai Tenth People’s Hospital, Tongji University School of Medicine, Shanghai, China; ^4^ Department of Cardiology, Ruijin Hospital, Shanghai Jiaotong University School of Medicine, Shanghai, China; ^5^ Department of Anesthesiology, Qilu Hospital, Cheeloo College of Medicine, Shandong University, Jinan, China; ^6^ School of Medicine, Cheeloo College of Medicine, Shandong University, Jinan, China; ^7^ Department of Cardiology, Johns Hopkins School of Medicine, Baltimore, MD, United States; ^8^ Department of Anesthesiology and Critical Care Medicine, Johns Hopkins University School of Medicine, Baltimore, MD, United States; ^9^ Clinical Trial Institution, Nanjing Drum Tower Hospital, Affiliated Hospital of Medical School, Nanjing University, Nanjing, Jiangsu, China

**Keywords:** FFAR2, FFAR3, single-cell atlas, heart, human, mice

## Abstract

**Introduction:**

The gut microbiota metabolite, short-chain fatty acids (SCFAs), can protect against multiple cardiovascular diseases, while the molecular targets and underlying mechanisms need to be elucidated. One of the primary mechanisms of SCFA benefits was the direct activation of a group of G-protein-coupled receptors (GPCRs), termed free fatty acid receptors (FFARs), the FFAR2 (GPR43), and FFAR3 (GPR41). At present, the distribution of FFAR2/3 in cardiac cells has not been entirely clarified.

**Methods:**

Using 18 public single-cell RNA-seq and single-nuclear RNA-seq data of human and mouse hearts, we illustrate the entire atlas of *FFAR2/3* distribution in different regions and cell types in normal and infarcted hearts.

**Results and discussion:**

We present the atlas of *FFAR2/3* in the whole human body, normal and infarcted hearts at single-cell resolution. We also illustrated the entire atlas of *FFAR2/3* in normal/ischemic hearts of newborn and adult mice by combining public and newly built sc/snRNA-seq datasets. These findings provide valuable information on the possible effect of SCFAs via FFAR2/3 in the heart and valuable references for future studies.

## Introduction

Trillions of microorganisms reside in the human intestine forming the gut microbiota, which is a crucial element for host health and homeostasis (1, 2). The gut microbiota communicates with the host via gut microbial metabolites, particularly short-chain fatty acids (SCFAs) (3). SCFAs, the main product of dietary fiber fermentation by gut microbiota, are organic fatty acids with a carbon chain length of six or fewer (acetate (C2), propionate (C3), and butyrate (C4)), which can be absorbed into the bloodstream and play important roles in various physiological processes such as metabolism, gut barrier function, immune regulation, and inflammation (4).

The gut microbiome (5–7) and their metabolites, SCFAs, have been shown to have multiple effects on cardiovascular physiologic processes (8–10) and diseases, including atherosclerosis, hypertension, atrial fibrillation, myocardial infarction (MI), cardiac hypertrophy, and heart failure (2, 11–14). Accumulating evidence implicated that SCFAs benefits mainly rely on the agonistic effect on G-protein-coupled receptors (GPCRs), histone deacetylases (HDACs) inhibition, and its utilization as an energy source, all of which have not been entirely clarified and need further investigation (2).

GPCRs, also known as 7 transmembrane domain receptors (7TMRs) (15, 16), translate diverse extracellular stimulations (ions, small molecules, peptides, and proteins) into intracellular signals, serve as key regulators of a variety of intracellular responses (15, 17–19), and are the primary targets of approximately 35% of all small molecule drugs currently approved by the Food and Drug Administration (FDA) (20). The GPCRs that can be directly activated by SCFAs are termed free fatty acid receptors (FFARs), including FFAR2 (GPR43) and FFAR3 (GPR41), playing a crucial role in connecting the human gut and heart (21).

**Figure 1:**
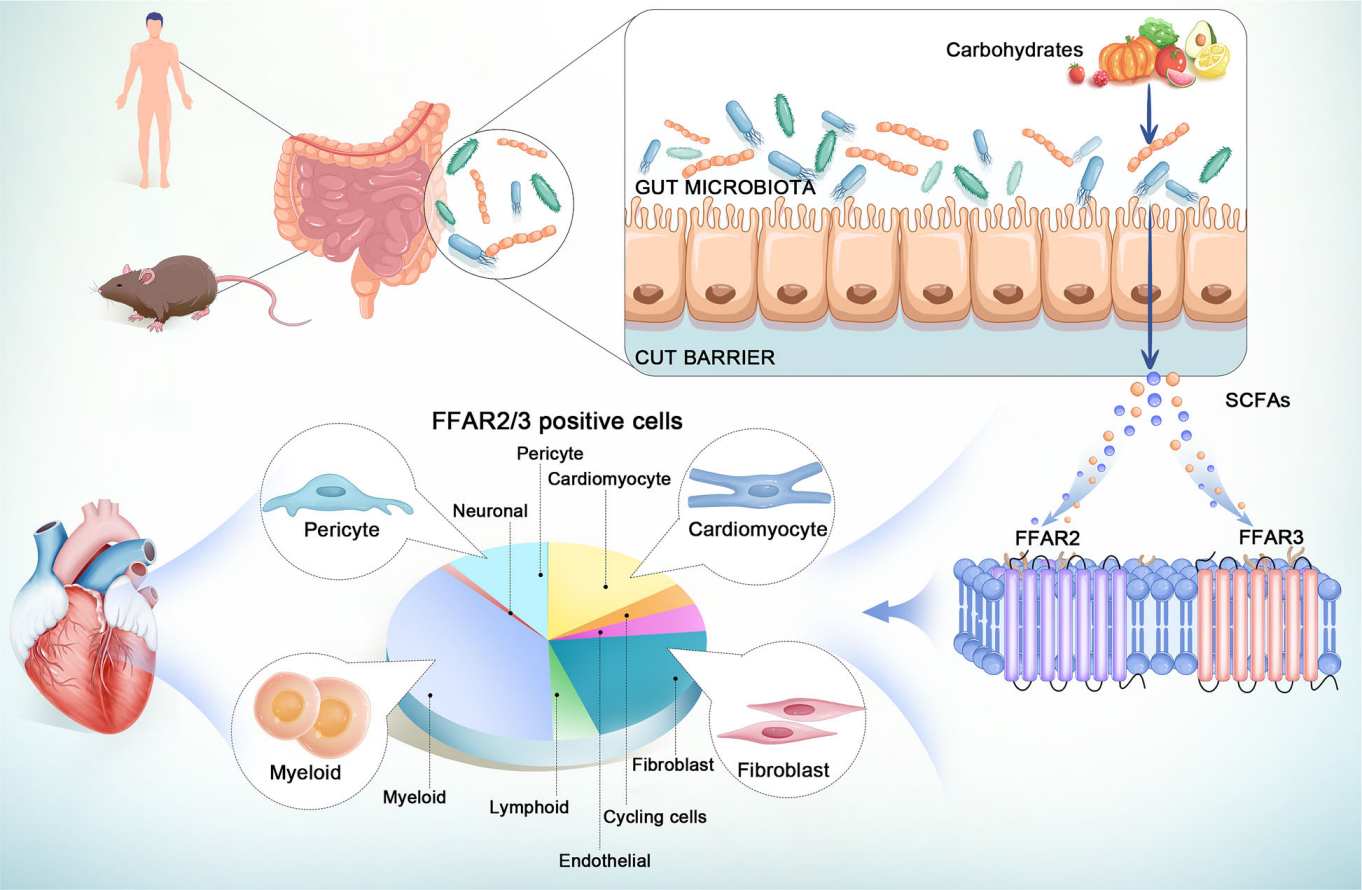
General design and result of the study.

However, because of the instability of the GPCRs and the lack of reliable antibodies, the heterogeneous distribution of FFAR2 and FFAR3 across multiple cell types in the heart remains to be elucidated in order to develop novel therapeutics (5, 22, 23). Single-cell (or nuclear) RNA sequence (sc/snRNA-seq) technology can reveal cell heterogeneity (24), cell subtype-specific expressed genes and disease-related cell state regulation (25). In this study, we present the atlas of *FFAR2/3* in the human body, especially in the normal and infarcted hearts at single-cell resolution, based on published data of single-cell and nuclear RNA sequence of human tissue (**Figure 1**). The mouse, whether a newborn or adult, is one of the most adopted model animals for biomedical research. It has been demonstrated the existence of regenerative capacity in neonatal mouse hearts, which is lost seven days after birth (26, 27). So we illustrate the entire atlas of *FFAR2*/*3* in normal/ischemic hearts of newborn and adult mice by combining public and newly built sc/snRNA-seq datasets (**Figure 1**). We believe our findings will set a foundation for further exploration of mechanisms for SCFA in the heart.

## Results

### Expression of *FFAR2/3* in the human heart

**Figure 2:**
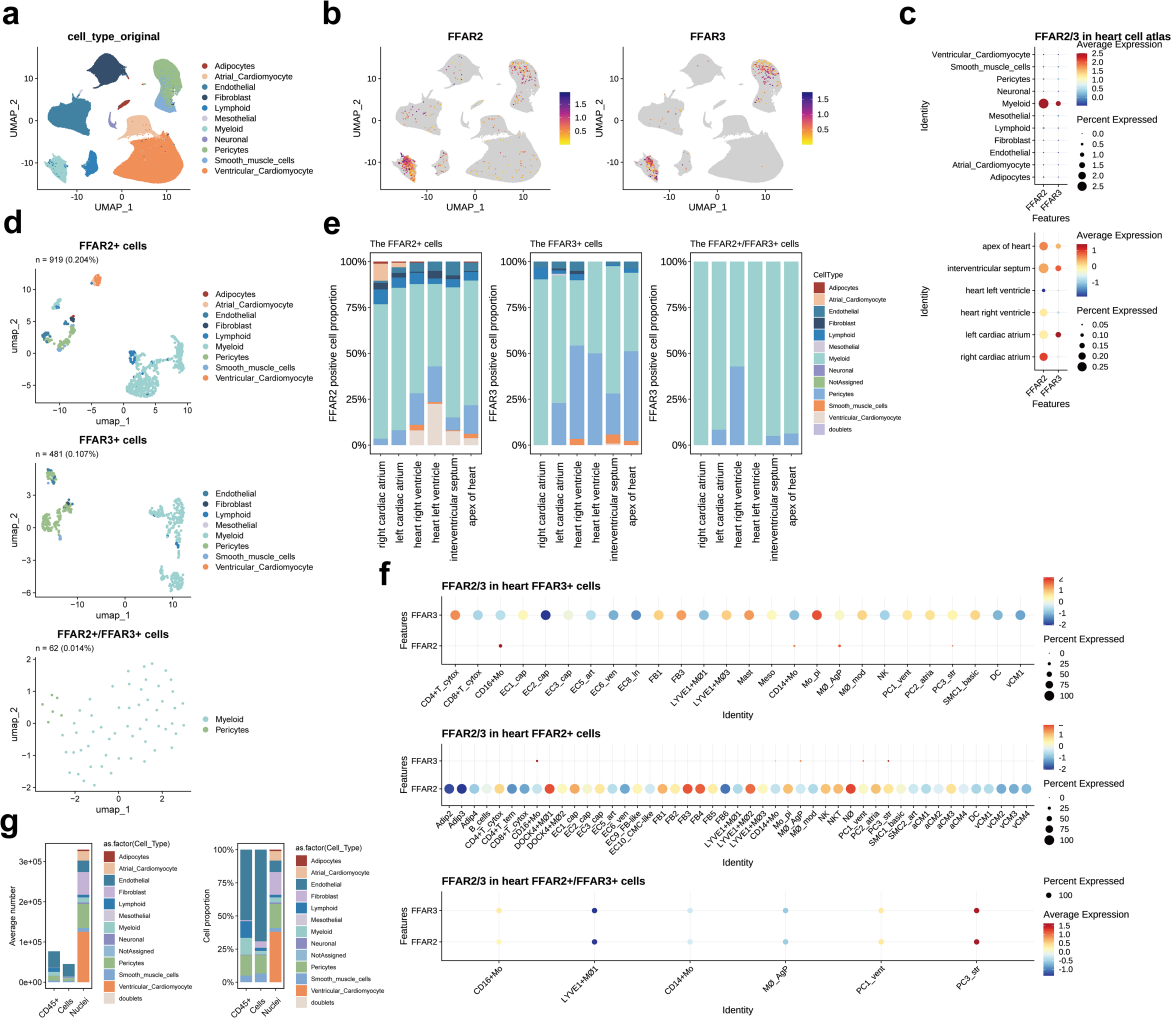
*FFAR2/3* distribution in the human heart. **(a)** UMAP embedding of the heart cell atlas (hca) dataset (n = 451,513 cells). **(b)** *FFAR2/3* expression in the UMAP plot. **(c)** *FFAR2/3* expression across multiple cell types and distinct cardiac regions. **(d)** UMAP of *FFAR2* ^+^, *FFAR3* ^+^, and *FFAR2* ^+^/*FFAR3* ^+^ cells in the human heart. **(e)** Proportion of *FFAR2/3* positive cell types in distinct cardiac regions. **(f)** Dot plot showing the expression level of *FFAR2* and *FFAR3* in *FFAR2* ^+^ and *FFAR3* ^+^ cells. **(g)** Proportion of *FFAR2/3* positive cell types in distinct sequence strategy. B_cells, B cells; CD4^+^ T_cytox, CD4^+^ cytotoxic T cells; CD4^+^ T_tem, effector-memory CD4^+^ T cells; CD8^+^ T_cytox, CD8^+^ cytotoxic T cells; CD8^+^ T_tem, CD8^+^ effector-memory T cells; CD16^+^ Mo, CD16^+^ monocytes; DOCK4^+^ MΦ1–2, DOCK4^+^ macrophage sets 1–2; EC1/2/3_cap, capillary ECs; EC4_immune, immune-related ECs; EC5_art, arterial ECs; EC6_ven, venous ECs; EC7_atrial, atria-enriched ECs; EC8_ln, lymphatic ECs; EC9_FB-like, ECs with FB features; EC10_CM-like, ECs with cardiomyocyte features; IL17RA^+^ Mo, IL17RA^+^ monocytes; LYVE1^+^ MΦ1–3, M2-like, LYVE1^+^ macrophages sets 1–3; CD14^+^ Mo, CD14^+^ monocytes; Mo_pi, pro-inflammatory monocytes; MΦ_AgP, HLA class II antigen-presenting macrophages; MΦ_mod, monocyte-derived macrophages; NK, natural killer; NKT, natural killer T cells; NΦ, neutrophils; PC1_vent, ventricle-enriched pericytes; PC2_atrial, atria-enriched pericytes; PC3_str, stromal pericytes; PC4_CM-like, pericytes with cardiomyocyte features; SMC1_basic, basic SMCs; SMC2_art, arterial SMCs; DC, dendritic cells.

In the “All-Cells of the adult human heart” (the human cell atlas, hca) dataset, consisting of 486,134 cardiac tissue cells, there were 1,030 (0.21%) *FFAR2* ^+^ cells and 525 (0.11%) *FFAR3* ^+^ cells. We did not change Dr. Teichmann’s annotation results of cell types and subtypes (24). As a result, most of these *FFAR2/3* ^+^ cells were myeloid cells and pericytes, distributed across the whole heart (**Figures 2a-d**, **Supplementary Figure S1**). Moreover, the *FFAR2*/*FFAR3* double-positive cells were only observed in myeloid and pericytes (**Figure 2d**, **Supplementary Figure S1**). In particular, *FFAR3* ^+^ endothelial was not found in the left ventricle, while *FFAR3* ^+^ pericytes were not found in the right atrium (**Figure 2e**). The expression of *FFAR2*/*3* ^+^ in the PC3_str, a potential transitional state between pericytes and endothelial cells (ECs) (24), indicates the likely effect of SCFA in endothelial differentiation (**Supplementary Figure S1**). The SCFA might also play a role in smooth muscle cell (SMC) development due to the expression of *FFAR2*/*3* on SMC1_basic, representing an immature SMC subtype (**Supplementary Figure S1**).

The *FFAR2* ^+^ cardiomyocytes (n = 70, 0.014% in all cells of the hca dataset) were located in all of the heart with equal distribution in the atrium (12 in 23,248 cells, 0.051%) and ventricle (58 in 64510 cells, 0.046%). Very few *FFAR3* ^+^ cardiomyocytes (n = 1, 0.000206%) were found in the interventricular septum (not shown in the figure). Notably, no *FFAR2* ^+^ mesothelial cell, *FFR3* ^+^ adipocyte, *FFAR3* ^+^ atrial cardiomyocyte, or *FFAR2*/*3* ^+^ neuronal cell was observed (**Figure 2d**, **Supplementary Figure S1**).

We then assessed the expression level of *FFAR2* and *FFAR3* in each subcluster of cell types via the dot plot (**Figure 2f**). The *DOCK4* ^+^ MΦ1 (*DOCK4* ^+^ macrophage sets 1), NΦ (neutrophils), FB3 (*PTX3*, *OSMR* and *ILST6*; cytokine receptors enriched) (24), FB4 (*POSTN*, *TNC*, *FAP*; responsive to TGFβ signaling) (24), and *LYVE1* ^+^ MΦ2 (*LYVE1* ^+^ macrophages sets 2) had a higher level of *FFAR2* than other cell subclusters. In contrast, the Mo_pi (pro-inflammatory monocytes), *CD4* ^+^ T_cytox (*CD4* ^+^ cytotoxic T cells), FB3, and Mast cells had higher levels of *FFAR3* expression. For the double-positive cells, the PC3_str has the highest level of *FFAR2* and *FFAR3*, followed by the PC1_vent and *CD16* ^+^ Mo (**Figure 2f**, bottom panel).

To explore the characteristics of *FFAR2*/*3* ^+^ cells, we calculated specifically upregulated genes (marker genes) in the *FFAR2*/*3* ^+^ cells compared to the *FFAR2* ^-^/*3* ^-^ cells (**Supplementary Table S1**). These marker genes were mainly derived from immune cells (myeloid and lymphoid cells) and mural cells (pericytes and SMC) (**Supplementary Figures S2a, b**). Multi types of heat shock proteins were observed in the *FFAR2*/*3* ^+^ myeloid and cardiomyocytes (**Supplementary Figure S2c**). Gene ontology (GO) annotation revealed the upregulation of the cytoplasmic protein synthesis and cellular respiration in *FFAR2*/*3* ^+^ cells (**Supplementary Figures S2d-f**, **Supplementary Table S2**). Weighted correlation network analysis (WGCNA) revealed active protein synthesis and energy metabolism in *FFAR2*/*3* ^+^ cells (**Supplementary Figure S3**) and immune regulation function in *FFAR3* ^+^ monocytes (**Supplementary Figures S3** and **S4**, **Supplementary Table S3**).

### Expression of *FFAR2/3* in the human heart with MI

**Figure 3:**
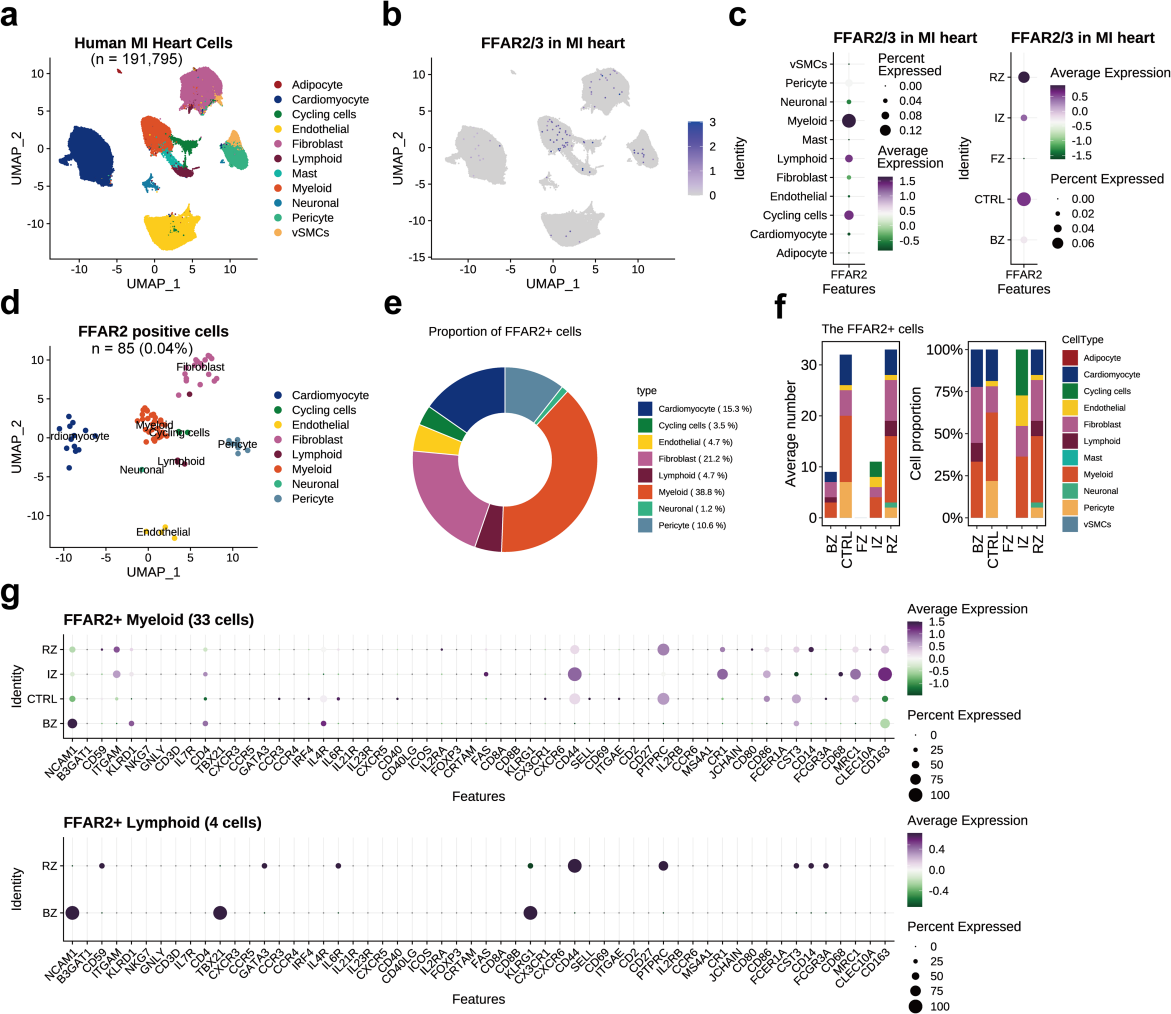
*FFAR2/3* distribution in the human myocardial infarction heart. **(a)** UMAP embedding of the human myocardial infarction heart (n = 191,795 cells). **(b)** *FFAR2* expression in the UMAP plot. **(c)** *FFAR2* expression across multiple cell types and distinct cardiac regions. **(d)** UMAP of *FFAR2* ^+^ cells. **(e)** circle plot showing the proportion of each cell type in *FFAR2* ^+^ cells. **(f)** Proportion of *FFAR2* ^+^ cell types in distinct cardiac regions. **(g)** Dot plot showing the immune cell marker gene expression level in *FFAR2* ^+^ cells of distinct cardiac infarction regions. RZ, remote zone, the unaffected left ventricular myocardium; BZ, border zone; IZ, ischaemic zone; FZ, fibrotic zone, human heart specimens at later stages after myocardial infarction; CTRL, control samples from non-transplanted donor hearts.

The proportion of *FFAR2* ^+^ cells and *FFAR3* ^+^ cells in normal heart hca dataset was much higher than that in the myocardial infarction datasets, which could be explained by the adoption of CD45+ cell enrichment method in hca dataset, leading to more captured myeloid cells (**Figure 2g**). In the human heart MI (the spatial multiomic atlas, sma) dataset consisting of 191,795 cells (**Figure 3a**), there were 85 (0.04%) *FFAR2* ^+^ cells, and no *FFAR3* ^+^ cells were found (**Figures 3b-d**). The immune cells and cycling cells have higher levels of *FFAR2* than other types of cells (**Figure 3c**). The myeloid occupied most *FFAR2* ^+^ cells (n=33, 38.82%), followed by the fibroblast (n=18, 21.18%) and cardiomyocyte (n=13, 15.29%) (**Figures 3d, e**). The proportion of FFAR2^+^ cells in ventricular cardiomyocytes was 0.020% (13 in 64,510 ventricular cardiomyocytes) since all the sma dataset samples come from cardiac ventricles (25).

Most of the *FFAR2* ^+^ cells came from control samples (CTRL) and the remote zone (RZ, the unaffected left ventricular myocardium) but were not detected in the fibrotic zone (FZ) (**Figures 3c, f**). An enlarged proportion of fibroblasts was observed in the myocardial infarction heart, which differed from the normal heart (**Figures 3e, f**). This could not be explained by the exclusion of atrial tissue in the sma dataset or the existence of the border zone (BZ) and ischemic zone (IZ) (**Figure 3f**). However, we noticed a decreased proportion of pericytes in RZ when compared to the CTRL samples from non-transplanted donor hearts, implying the impact of MI was across the entire heart and including the remote cardiac tissue. The existence of *FFAR2* ^+^ and *FFAR3* ^+^ cells in human myocardial infarction tissue, by immunofluorescent co-staining for FFAR2/3 and canonical marker genes of monocytes and macrophages (CD14, CX3CR1, and CD68), indicating the existence of FFAR2/3 positive monocytes and macrophages (**Supplementary Figure S5**).

The *FFAR2* ^+^ cell marker genes were primarily expressed on myeloid cells, similar to those in normal hearts (**Supplementary Table S4**, **Supplementary Figure S6a**). The top marker genes of *FFAR2* ^+^ myeloid were *CD55* and *CYBB*, which involved in complement cascade regulation and bacteria killing (**Supplementary Figure S6b**). On the other hand, the marker genes were annotated into calcium ion metabolism by GO enrichment analysis (**Supplementary Figure S6b**, **Supplementary Table S5**). Among the *FFAR2* ^+^ myeloid cells, the marker genes of the IZ part were recognized to be involved in extracellular matrix/structure organization (**Supplementary Figure S6c**). We also checked the expression of canonical immune cell type marker genes in different cardiac regions, finding the enrichment of *NCAM1* (neural cell adhesion molecule 1, also known as *CD56*) on both lymphoid and myeloid cells in the border zone (**Figure 3g**).

The top marker genes of *FFAR2* ^+^ fibroblasts were *RTN3*, which is expressed in neuroendocrine tissues and acts as an inhibitor of amyloid-beta, and *SORCS1*, which is strongly expressed in the central nervous system (**Supplementary Figure S6d**). WGCNA for highly variable genes in *FFAR2* ^+^ cells found the enrichment of microtubule anchoring and mitochondrion organization for *FFAR2* ^+^ fibroblasts (**Supplementary Figure S7**, **Supplementary Table S6**).

### Expression of *FFAR2/3* across human organs

**Figure 4:**
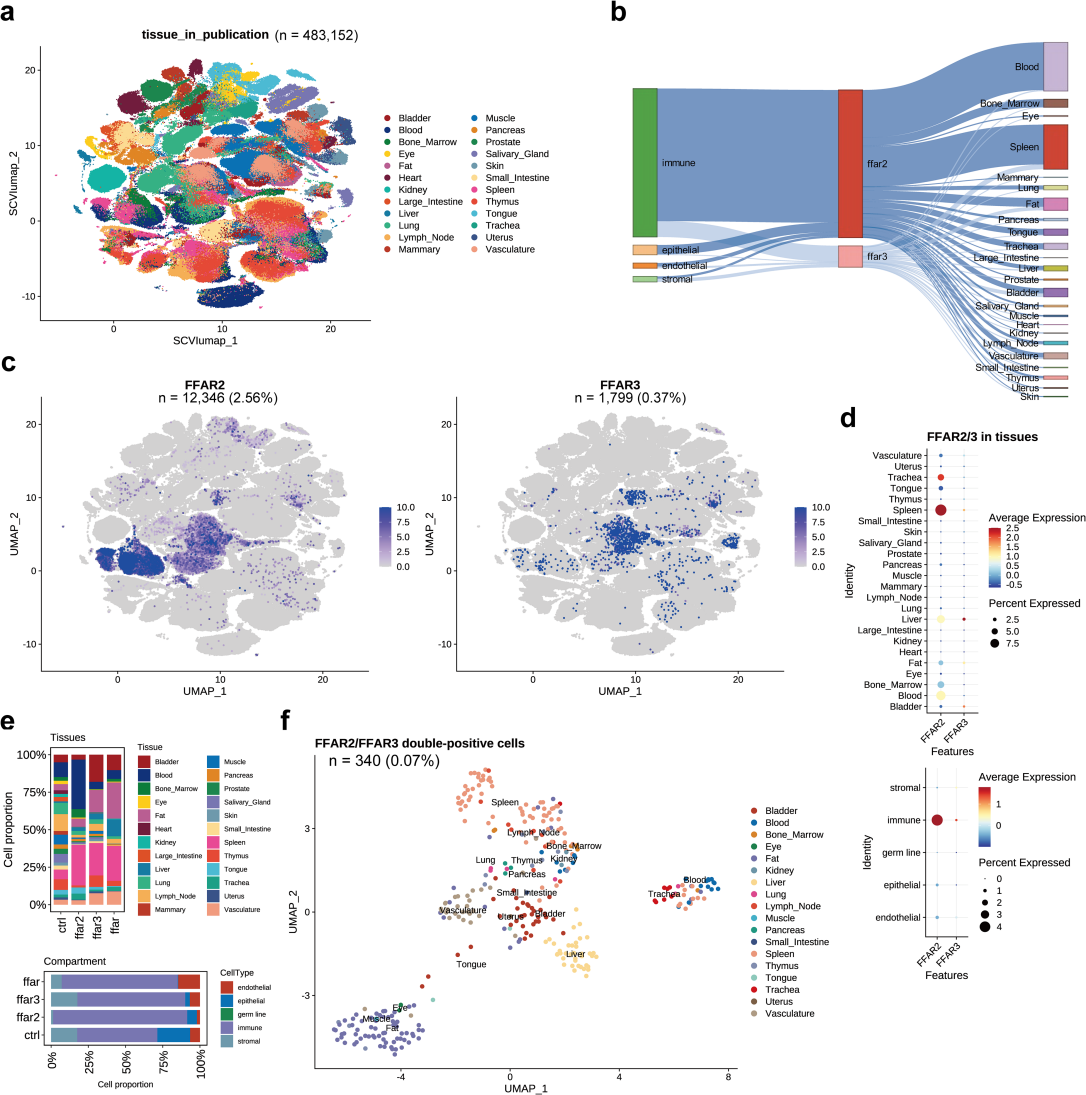
*FFAR2/3* distribution in human organs. **(a)** UMAP embedding of the tsc dataset (n = 483,152 cells). **(b)** Illustration of *FFAR2/3* expression across multiple cell types and tissues. **(c)** UMAP embedding of *FFAR2/3* gene expression. **(d)** Expression level *FFAR2/3* across 24 human tissues (upper panel) and five cell types (lower panel). **(e)** Tissue (upper panel) and compartment (lower panel) distribution of *FFAR2* ^+^, *FFAR3* ^+^, and *FFAR2* ^+^/*FFAR3* ^+^ cells. **(f)** UMAP embedding of *FFAR2* ^+^/*FFAR3* ^+^ cells of the tsc dataset (n = 340 cells, 0.07%).

We also explored FFAR2/3^+^ cells in whole human body via the “TS_All_Cells” (tsc) dataset. There were 12,346 (2.56%) *FFAR2* positive cells and 1,799 (0.37%) *FFAR3* positive cells in the tsc human dataset consisting of 483,152 cells, and most of the *FFAR2/3* ^+^ cells were immune cells distributed in spleen and blood (**Figures 4a–c**, **Supplementary Table S7**). Spleen and trachea have the highest level of *FFAR2*, while the liver and bladder have the highest level of *FFAR3* (**Figure 4d**). Spleen-derived cells occupied the most significant proportion of both *FFAR2* ^+^, *FFAR3* ^+^, and co-expression cells (**Figure 4e**, upper panel). Interestingly, *FFAR2* ^+^ cells are also distributed in blood. In contrast, there were many fat-derived cells for *FFAR3* ^+^ and co-expression cells. We also found more stromal cells in *FFAR3* ^+^ cells than the *FFAR2* ^+^ and co-expression cells and more endothelial in co-expression cells than in other groups (**Figure 4e**, lower panel). There were 340 (0.07%) co-expression cells in the tsc dataset, most of which were spleen, fat, liver, vasculature, and bladder cells (**Figure 4f**). In the heart, only 6 *FFAR2* ^+^ cells and 2 *FFAR3* ^+^ cells detected (not presented in the figure).

### Expression of *FFAR2/3* in the mice heart

**Figure 5:**
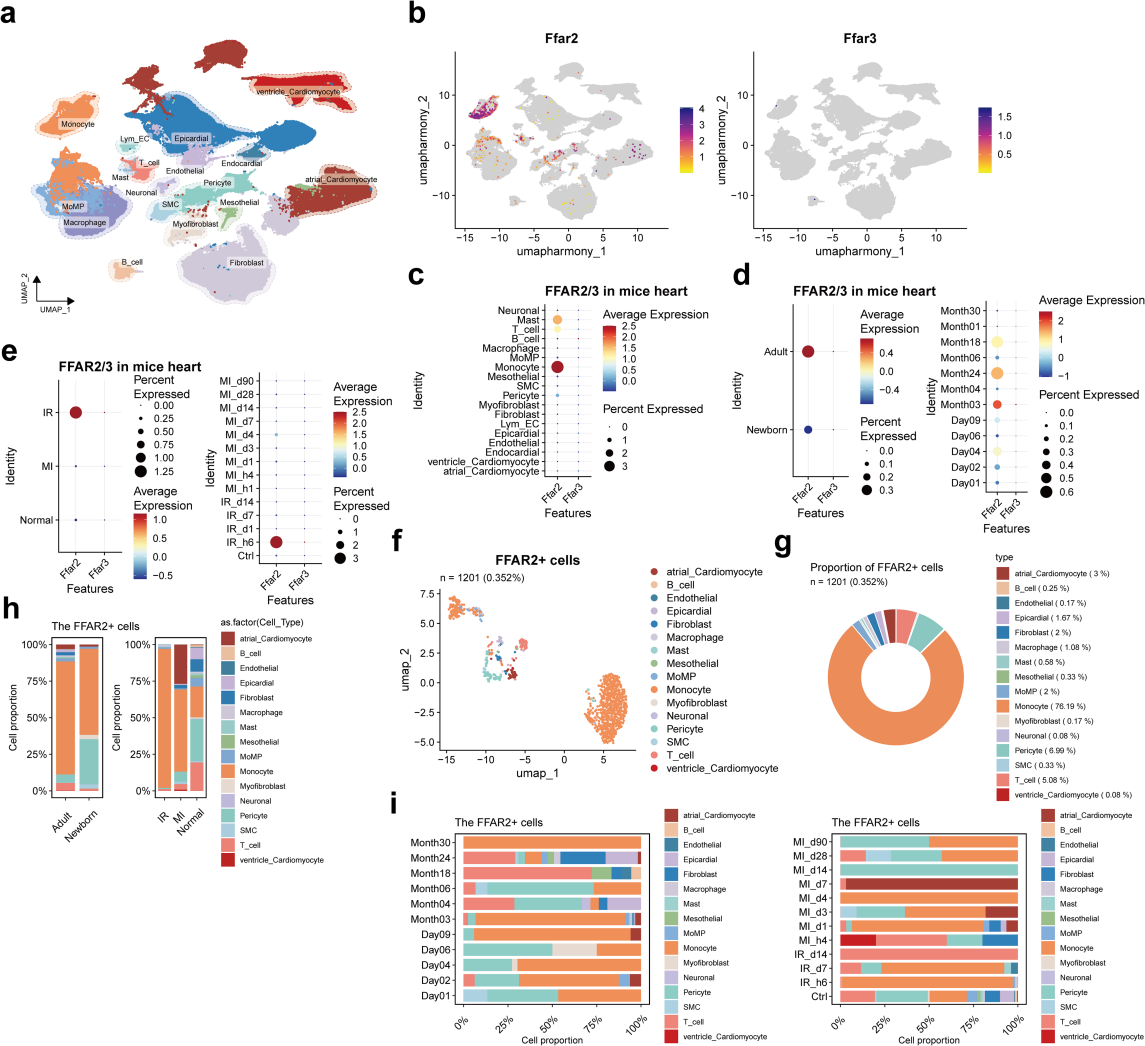
*FFAR2/3* distribution in mice heart. **(a)** UMAP of cell types in the heart of mice. There were 283,704 cells integrated in the combined atlas. **(b)** *FFAR2/3* expression in the UMAP plot. **(c)** *FFAR2/3* expression across multiple cell types. **(d)** *FFAR2/3* expression across development stages and ages. **(e)** *FFAR2/3* expression across multiple diseases and different follow-up time points. **(f)** UMAP of *FFAR2* ^+^ cells in mice hearts. **(g)** circle plot showing the proportion of each cell type in *FFAR2* ^+^ cells. **(h)** Proportion of each cell type for *FFAR2* ^+^ cells. **(i)** Proportion of ages (left panel) and follow-up time points (right panel) for *FFAR2* ^+^ cells.

We combined 15 public single-cell RNA sequence datasets and 1 dataset produced by our team, building an integrated dataset of mice heart tissue that consisted of 341,125 cells (**Supplementary Figures S8**, **S9**, **Figure 5a**, **Supplementary Table S8**), among which 8.79% (29,981 cells) were from newborn mice and the other 91.21% (311,144 cells) were from adult mice (**Figure 5a**). Only one *Ffar3* ^+^ cell was observed (**Figure 5b**). The *FFAR2* was expressed with higher proportion and levels in monocyte and Mast cells (**Figure 5c**), adult individuals (**Figure 5d**), and ischemic-reperfusion (I/R) injury cardiac tissue, particularly 6 hours after I/R injury (**Figure 5e**).

There were 1201 (0.352%) Ffar2^+^ cells detected, 5.66% (68 cells) were in the newborn group, and 94.34% (1133 cells) were in the adult group (**Figure 5f**). In the *Ffar2* ^+^ cells, 76.19% were monocytes, followed by pericyte (6.99%), T cells (5.08%), and macrophage (MoMP (monocyte-derived macrophage) and resident macrophage, 3.08%) (**Figure 5g**).

The decreased *Ffar2* ^+^ pericyte proportion in the I/R and MI group was observed, which is consistent with the result in human MI data (**Figures 3f**, **5h**). The large proportion of *Ffar2* ^+^ monocyte in 6 hours post-I/R injury and four days post-MI was reasonable due to the inflammatory response and monocyte infiltration (**Figure 5i**). The individuals at the age of day 4 were neonatal mice who received MI injury three days ago, while those at the age of day 9 were adult mice who received MI one day ago. The large proportion of *Ffar2* ^+^ monocytes at 3-day post-MI in neonatal mice and 1-day post-MI in adult mice indicate a distinction between neonatal and adult mice for *Ffar2* ^+^ monocyte response after MI injury.

The differentially expressed genes (DEGs) compared to non-*Ffar2* cells are distributed in monocytes, MoMP, and macrophages (**Supplementary Figure S10a**). GO enrichment not only found the human *Ffar2* cells enriched biological process—cytoplasmic translation, but also the process of translation within synapse (**Supplementary Figure S10b**).

## Discussion

In this study, we explored the single-cell level distribution of *FFAR2* and *FFAR3* in human and mice cardiac tissue based on several public RNA-sequence datasets. We quantify the cellular composition and marker genes of *FFAR2* ^+^, *FFAR3* ^+^, and double-positive cells for normal and injured cardiac tissue. Co-expressed gene expression modules and top hub genes ranked by eigengene-based connectivity (kME) were also calculated, representing the potential downstream mediators of SCFA-FFAR2/FFAR3 biological effects under physiological and/or disease conditions.

In normal human hearts, the *FFAR2/3* is expressed mainly in myeloid cells, followed by pericytes and fibroblasts which indicated the immune regulation, endothelial differentiation, and smooth muscle cell (SMC) development are main effects of SCFA and FFAR2/3. The *DOCK4* ^+^ macrophage, neutrophils, FB3 (cytokine receptors enriched), FB4 (responsive to TGFβ signaling), and *LYVE1* ^+^ macrophages had higher level of *FFAR2*, while pro-inflammatory monocytes, *CD4* ^+^ cytotoxic T cells, FB3, and Mast cells had higher levels of *FFAR3.* Specific upregulated genes in *FFAR2/3* cells were annotated into cytoplasmic protein synthesis and cellular energy metabolism. Multi types of heat shock proteins were observed in the *FFAR2*/*3* ^+^ myeloid and cardiomyocytes.

In human myocardial infarction, the myeloid occupied most *FFAR2* ^+^ cells, followed by the fibroblast and cardiomyocyte. An enlarged proportion of fibroblasts and decreased proportion of pericytes were observed in the myocardial infarction. Most of the *FFAR2* ^+^ cells came from the unaffected remote zone left ventricular myocardium, and no *FFAR2* ^+^ cells were observed in the fibrotic zone, even though the total cell number of the fibrotic zone was not significantly less than other samples. The more extensive set of *FFAR2* ^+^ cells in control and RZ than injured cardiac tissue implied more substantial immunoregulation effects of SCFA in these regions. The *NCAM1* (neural cell adhesion molecule 1, also known as *CD56*) was highly expressed on both lymphoid and myeloid cells in the border zone. The *FFAR2* ^+^ myeloid cells are related to calcium ion metabolism and complement cascade, while the IZ part of *FFAR2* ^+^ myeloid cells were recognized to be involved in extracellular matrix/structure organization. The enrichment of microtubule anchoring and mitochondrion organization were observed for *FFAR2* ^+^ fibroblasts in human MI.

There is a 5-fold difference between the proportion of *FFAR2* ^+^ cells in the normal and infarction human heart dataset (0.21% vs 0.04%). This difference may partially come from the difference in RNA sequence strategy. The hca dataset consists of three parts, the CD45^+^ cells enrichment data, the single-cell RNA-sequence (scRNA-seq, cardiomyocytes were excluded in this strategy) data, and the single-nucleus RNA-sequence (snRNA-seq, cardiomyocytes were included) data (24). In contrast, the scRNA-seq strategy was not adopted in the MI sma dataset (25). So, there should be a larger proportion of cardiomyocytes in the sma dataset compared to the hca dataset. This was in accordance with the *FFAR2* distribution (**Figures 2c** and **3c**). Namely, among the *FFAR2* ^+^ cells, cardiomyocytes occupied 6.8% in hca dataset and 15.29% in the sma dataset. Another potential reason for the 5-fold difference is the decrease of FFAR2 in peripheral venous leukocytes of myocardial infarction patients, reported by Ruan J, etc. (28).

In cardiac tissue, an association between gut microbiota depletion and significant reductions in the proportion of myeloid cells and SCFAs had been reported (29). The FFAR3 may necessary for the benefits of propionate, one of the SCFA, myocardial ischemia-reperfusion injury (30). Despite the absence of *FFAR3* ^+^ cells in the sma dataset, which may come from both low proportion and cell capture limitations, the existence of *FFAR2* ^+^ and *FFAR3* ^+^ monocytes and macrophages cells in human myocardial infarction tissue has been proved by immunofluorescent co-staining in this study.

During myocardial infarction (MI), ischemic injury initiates the release of damage-associated molecular patterns (DAMPs) from the infarcted myocardium (31). This triggers the infiltration of blood neutrophils (32), leading to recruitment of peripheral CCR2^+^ monocytes which differentiate into macrophages and help debride the cardiac wound but can also contribute to tissue injury (33–35). Many of the DAMPs serve as ligands for pattern recognition receptors (PPRs), including Toll-like receptors (TLRs), NOD-like receptors (NLRs), and complement receptors (31). While targeting complement cascade was successful in attenuating animal ischemic cardiac injury, translation for human populations with myocardial infarction was unsuccessful and failed to reduce infarct size (36). In our study, the *FFAR2* ^+^ myeloid cells of human infarcted myocardium are enriched with calcium ion metabolism and complement cascade, while the IZ part of *FFAR2* ^+^ myeloid cells were recognized to be involved in extracellular matrix/structure organization. This indicates the involvement of SCFA in complement reaction after ischemic injury which might be important for ischemic cardiac injury pathophysiology. The expression of *FFAR3* in pro-inflammatory monocyte revealed that SCFA might participate in the regulation of monocytes recruitment and differentiation.

Resident cardiac macrophage lacking CCR2 (CCR2**^–^**), originates from the yolk sac and fetal liver during development, and is prenatally seeded and maintained by self-renewal (35). CCR2**^–^** macrophages are further divided by expression of major histocompatibility complex (MHC) class II, as MHCII^hi^ and MHCII^lo^ populations, the latter of which are characterized as T cell immunoglobulin and mucin domain containing 4 (TimD4^+^), lymphatic vessel endothelial hyaluronan receptor 1 (Lyve1^+^), and folate receptor 2 (FolR2^+^) (37, 38). In the developing and homeostatic heart, self-renewing resident macrophages (CCR2^-)^ contribute to the formation and maintenance of the vasculature, electrical conduction, and phagocytosis of dysfunctional organelles in the myocardium. In the ischemic injury and reperfusion injury, resident macrophages sense injury, which also triggers macrophage proliferation (39), can counterbalance inflammation through the inhibition of monocyte recruitment, and serve as cardio protectors in response to heart injury (39, 40). The expression of *FFAR2*/*3* in Lyve1^+^ macrophage, PC3_str, and SMC1_basic revealed the function SCFA in formation and maintenance of the vasculature via multiple cell types, which needs to be clarified in the future.

The *FFAR2* ^+^ fibroblasts in infarcted myocardium were enriched with *RTN3*, which is expressed in neuroendocrine tissues and acts as an inhibitor of amyloid-beta (Aβ). Aβ has been suggested to play a role in the pathogenesis of IHD and cerebral IRI (41). It can change the transcriptional profile of endothelium and cardiomyocyte that related to the ubiquitin-proteasome system, apoptosis, DNA damage and inflammation. The ischemic-reperfusion injury is associated with the increased production of some key transcription factors (AP-1, HIF-1α and NF-κB), cytokines (e.g., TNF-α, IL-1, IL-6, IL-8 and platelet-activating factor (PAF)), and increased intracellular Ca^2+^, all can stimulate the increase of Aβ (41). However, the relationship between fibroblast and Aβ in cardiac ischemic and reperfusion injury remains to be elucidated.

We found the enrichment of *NCAM1* (neural cell adhesion molecule 1, also known as *CD56*) on both lymphoid and myeloid cells in the border zone (**Figure 2g**). Even though the role of NCAM1 has not been explored in MI, the *NCAM1*-encoded protein plays a role in the expansion and migration of T lymphocytes, B lymphocytes, and natural killer (NK) cells, which play an essential role in immune reaction (42). It has been proposed as a potential diagnosis biomarker for MI (43). In our study, the enrichment of *NCAM1* in the MI heart was observed in both *FFAR2* ^+^ cells and the entire heart cells, either for mice or human datasets (**Supplementary Figure S11**). This implies the involvement of *NCAM1* in inflammatory response after cardiac injury and the immunoregulation function of SCFA via targeting lymphoid cells.

In summary, we found that *FFAR2* and *FFAR3* are mainly expressed in myeloid cells followed by pericytes and fibroblasts, in both human and mouse hearts indicating the indispensable role of SCFA in immune (and inflammation) regulation in the myocardium. Gene expression module analysis revealed that FFAR2/3 also involve various cellular biochemical processes. In human MI tissue, *FFAR2* and *FFAR3* may also regulate mitochondrial respiration. Our analysis highlights the *FFAR2* and *FFAR3* distribution in different cardiac tissue, cell types, and infarction areas, uncovering the potential effect and mechanisms of SCFAs via *FFAR2* and *FFAR3* in the heart, and provides a valuable reference for future studies.

## Methods

### Data acquisition and processing

For the analysis of human data, four processed single-cell RNA-seq datasets, the “TS_All_Cells” (tsc dataset, DOI: 10.1126/science.abl4896) (44), the “All-Cells of the adult human heart” (hca dataset, doi.org/10.1038/s41586-020-2797-4) (24), the spatial multiomic atlas data “All-snRNA-Spatial multi-omic map of human myocardial infarction” (snRNA of sma, doi.org/10.1038/s41586-022-05060-x) (25) were downloaded from the cellxgene database(https://cellxgene.cziscience.com/datasets). We did not change the cell annotation and quality control results of the hca, the sma, the tsc, and the aged mice tissue datasets since the authors have done great work and the processed data were provided. The ‘doublets’ and unrecognized cells (‘NotAssigned’) of the hca dataset have been excluded.

To get an integrated dataset for mice heart analysis, we searched the PubMed database, from the establishment to September 2024, for studies that have done single-cell or single-nucleus RNA-seq with mice heart tissue. The studies without reporting the RNA-seq method or do not have available sequence data (expression matrix or raw data) or that select cells for particular cell types were excluded. Count matrix data for the mice heart were downloaded from the GEO (GSE130699, GSE153480, GSE155882, GSE157244, GSE180821, GSE185265, GSE213337, GSE214611, GSE227088, GSE232466, and GSE247139 (PRJNA1035882)), ArrayExpress (E-MTAB-9816 and E-MTAB-9817), and (CRA005739) databases. These count matrix data were filtered and log-normalized, merged with the aged mice tissue datasets (doi.org/10.1038/s41586-020-2496-1) (45).

The count matrix data from other studies passed through quality control procedures with the Seurat R package (Version 4.4.0 & 5.0) (46, 47). In detail, cells were filtered out with gene numbers less than 200 and log10GenesPerUMI less than 0.8. The total cells were further filtered for the unique molecular identifier (UMI) (nUMI > 500), genes (200 < nGene), mitochondrial genes (mitoRatio < 20%), ribosomal genes (riboRatio <20%), and hemoglobin genes (hbRatio <5%). Single nuclei were further filtered for counts (nUMI > 500), genes (300 < nGene < 6,000), mitochondrial genes (mitoRatio < 5%), ribosomal genes (riboRatio < 5%), and hemoglobin genes (hbRatio < 5%) (25). The DoubletFinder package (48) (version 2.0.2) was applied to identify potential doublet. Batch effect correction was performed using Harmony (version 1.2.0) (49).

The integrated count matrice was passed for downstream analysis, including normalization (scaling factor = 10,000), building a shared nearest neighbor graph (SNN, with the first 30 harmony’s principal components), clustering (resolution = 1), and two-dimensional embedding (UMAP, with the first 30 harmony’s principal components). The Seurat object was overclustered (resolution = 2.0) and recognized based on published articles (24, 25).

Specific genes were visualized via the Seurat’s DotPlot, FeaturePlot, and RidgePlot function. The Sankey plot was constructed by the networkD3 package. Differential expression genes were calculated via the FindAllMarkers function of the Seurat package.

To gain a broader understanding of the cell type-specific transcriptional programs that are activated or repressed in the *FFAR2/3* positive cardiac tissue, we used weighted correlation network analysis (WGCNA) to study genes that are expressed in at least 5% of the cells of the whole dataset through the hdwgcna package (50–52). The function of co-expression gene modules was annotated by Gene Ontology (GO) terms with the enrichR and the GeneOverlap package. The enrichment test included the top 100 genes for each module. To explore the difference between *FFAR2/3* ^+^ cells and non-FFAR2/3 cells, the z-score of gene sets that are specifically highly expressed in *FFAR2/3* ^+^ cells (marker genes of *FFAR2*/*3* ^+^ cells) was calculated and plotted with the ArchR package (53, 54).

### Immunohistology

Human cardiac ventricle tissues were obtained from well-characterized patients undergoing elective coronary artery bypass graft surgery at the Ruijin Hospital, Shanghai Jiaotong University School of Medicine. Human tissue collection was conducted according to the Declaration of Helsinki and was approved by the institutional review board of the Ethics Committee of the Ruijin Hospital, Shanghai Jiaotong University School of Medicine. All patients gave written, informed consent before sample collection, as required. Full-thickness Atrial appendage biopsies, with the intentional exclusion of large epicardial fat deposits, were collected by the surgeon before cardiopulmonary bypass, fixed in formalin and embedded with paraffin for section and staining.

These formalin-fixed paraffin-embedded (FFPE) samples were cut into 4-μm sections, deparaffinized, and rehydrated. Heat-induced antigen retrieval was performed, and the sections were permeabilized with protease K (5ug/ml) for 10 min at room temperature. The sections were then blocked with either 10% normal rabbit serum or 3% BSA (the primary antibody is from rabbit and sealed with 10% rabbit serum, and the primary antibody from other sources is sealed with 3% BSA). Primary antibody incubation was performed overnight at 4 °C. After three washes in PBS (PH7.4), secondary antibody incubation was carried out at room temperature for 50 minutes. Antibodies adopted in this study were as follows: Anti-FFAR3/GPR41, bs-16076R, Bioss; Anti-FFAR2/GPR43, bs-13536R, Bioss; Anti-CD14, GB11254, Servicebio; Anti-CX3CR1, GB11861, Servicebio; Anti-CD68, GB113109, Servicebio. Nuclei were counterstained with DAPI (5 μg/ml for 8 min at room temperature). To remove lipofuscin and autofluorescence signal, tissues were incubated with TrueBlack Plus quencher (Biotium) for 10 min at room temperature. All the slides were scanned using a confocal scanning microscope.

## Code availability

The code for the study was available at https://github.com/Xiao851213/SCFA_FFAR/tree/Xiao851213-scfa_ffar.

## Data Availability

The datasets analyzed during the current study (the “TS_All_Cells” dataset, the “All-Cells of the adult human heart” dataset, the “All-snRNA-Spatial multi-omic map of human myocardial infarction” dataset, and then the aged mice tissue datasets) are available in the cellxgene database (https://cellxgene.cziscience.com/collections/e5f58829-1a66-40b5-a624-9046778e74f5, https://cellxgene.cziscience.com/collections/b52eb423-5d0d-4645-b217-e1c6d38b2e72, https://cellxgene.cziscience.com/collections/8191c283-0816-424b-9b61-c3e1d6258a77, https://cellxgene.cziscience.com/collections/0b9d8a04-bb9d-44da-aa27-705bb65b54eb). The Mice heart datasets are available in the GEO database with accession numbers GSE130699, GSE153480, GSE155882, GSE157244, GSE180821, GSE185265, GSE213337, GSE214611, GSE227088, GSE232466, and GSE247139; and the ArrayExpress database with accession numbers E-MTAB-9816 and E-MTAB-9817; and the GSA database with accession numbers CRA005739. All data generated or analyzed during this study are included in this published article (and its **Supplementary Information** files). Additional data that supports the findings of this study are available from the corresponding author upon reasonable request.
