# Supplementary material for: The single-cell atlas of short-chain fatty acid receptors in human and mice hearts
Source: Front Immunol. 2025 Apr 16;16:1538384. doi: 10.3389/fimmu.2025.1538384 (PMC12040890; doi:10.3389/fimmu.2025.1538384)
Supplement: Supplementary file 1 [file DataSheet1.docx]

**Supplemental Materials**

**The Single-cell Atlas of Short-chain Fatty Acid Receptors in Human and Mice Hearts**

## Author list

Xiaojun He^1,2#^, Qiang Wang^1#^, Qiang Long^2^, Yiming Zhong^2^, Zhaoxi Qi^2^, Yecen Zhang^2^, Lan Chang^2^, Bei Qian^2^, Shixing Huang^2^, Xinming Wang^2^, Xuemei Chen^3^, Feifei Li^4^, Xiaomei Yang^5,6,7^, Wei Dong Gao^8^, Zhizhao Song^1^*, Li Xu^9^*, Qiang Zhao^2^*.

^1^Cardiovascular Medical Center, Department of Cardiovascular Surgery, Nanjing Drum Tower Hospital, Affiliated Hospital of Medical School, Nanjing University, Nanjing, 210008, China

^2^Department of Cardiovascular Surgery, Ruijin Hospital, Shanghai Jiaotong University School of Medicine, Shanghai, China.

^3^Department of Anesthesiology, Shanghai Tenth People's Hospital, Tongji University School of Medicine, Shanghai, China.

^4^Department of Cardiology, Ruijin Hospital, Shanghai Jiaotong University School of Medicine, Shanghai, China.

^5^Department of Anesthesiology, Qilu Hospital, Cheeloo College of Medicine, Shandong University, Jinan, China.

^6^School of Medicine, Cheeloo College of Medicine, Shandong University, Jinan, China.

^7^Department of Cardiology, Johns Hopkins School of Medicine, Baltimore, MD, USA.

^8^Department of Anesthesiology and Critical Care Medicine, Johns Hopkins University School of Medicine, Baltimore, Maryland, USA.

^9^Clinical Trial Institution, Nanjing Drum Tower Hospital, Affiliated Hospital of Medical School, Nanjing University, Nanjing, 210008, China.

^#^These authors contribute equally to this study.

*Correspondence Authors

Qiang Zhao, email: [zq11607@rjh.com.cn](mailto:zq11607@rjh.com.cn); Li Xu, email: [iamxuli@163.com](mailto:iamxuli@163.com); Zhizhao Song, email: songzhizhao@outlook.com.

# Supplemental Table

## Supplemental Table 7

| **Organs** | **FFAR2^+^ cells** | **FFAR3^+^ cells** |
| --- | --- | --- |
| Bladder | 426(3.45%) | 299(16.62%) |
| Blood | 3988(32.30%) | 91(5.06%) |
| Bone_Marrow | 678(5.49%) | 10(0.56%) |
| Eye | 90(0.73%) | 7(0.39%) |
| Fat | 734(5.95%) | 298(16.56%) |
| Heart | 6(0.05%) | 2(0.11%) |
| Kidney | 21(0.17%) | 7(0.39%) |
| Large_Intestine | 14(0.11%) | 3(0.17%) |
| Liver | 332(2.69%) | 97(5.39%) |
| Lung | 323(2.62%) | 50(2.78%) |
| Lymph_Node | 217(1.76%) | 79(4.39%) |
| Mammary | 13(0.11%) | 1(0.06%) |
| Muscle | 106(0.86%) | 29(1.61%) |
| Pancreas | 188(1.52%) | 24(1.33%) |
| Prostate | 91(0.74%) | 12(0.67%) |
| Salivary_Gland | 143(1.16%) | 21(1.17%) |
| Skin | 17(0.14%) | 18(1.00%) |
| Small_Intestine | 45(0.36%) | 19(1.06%) |
| Spleen | 3325(26.93%) | 394(21.90%) |
| Thymus | 171(1.39%) | 124(6.89%) |
| Tongue | 496(4.02%) | 28(1.56%) |
| Trachea | 492(3.99%) | 26(1.45%) |
| Uterus | 51(0.41%) | 19(1.06%) |
| Vasculature | 379(3.07%) | 141(7.84%) |
| Total | 12346(100.00%) | 1799(100.00%) |

Supplemental Table 3 **| Distribution of** FFAR2/3^+^ cells across the human body.

The results were calculated from the “TS_All_Cells” dataset (https://cellxgene.cziscience.com/collections/e5f58829-1a66-40b5-a624-9046778e74f5) with the Seurat R package (v4.4.0).

Note: Data are presented as numbers (percentage of whole body FFAR2/3^+^ cells).

# Supplemental Figure

## Supplemental Figure 1


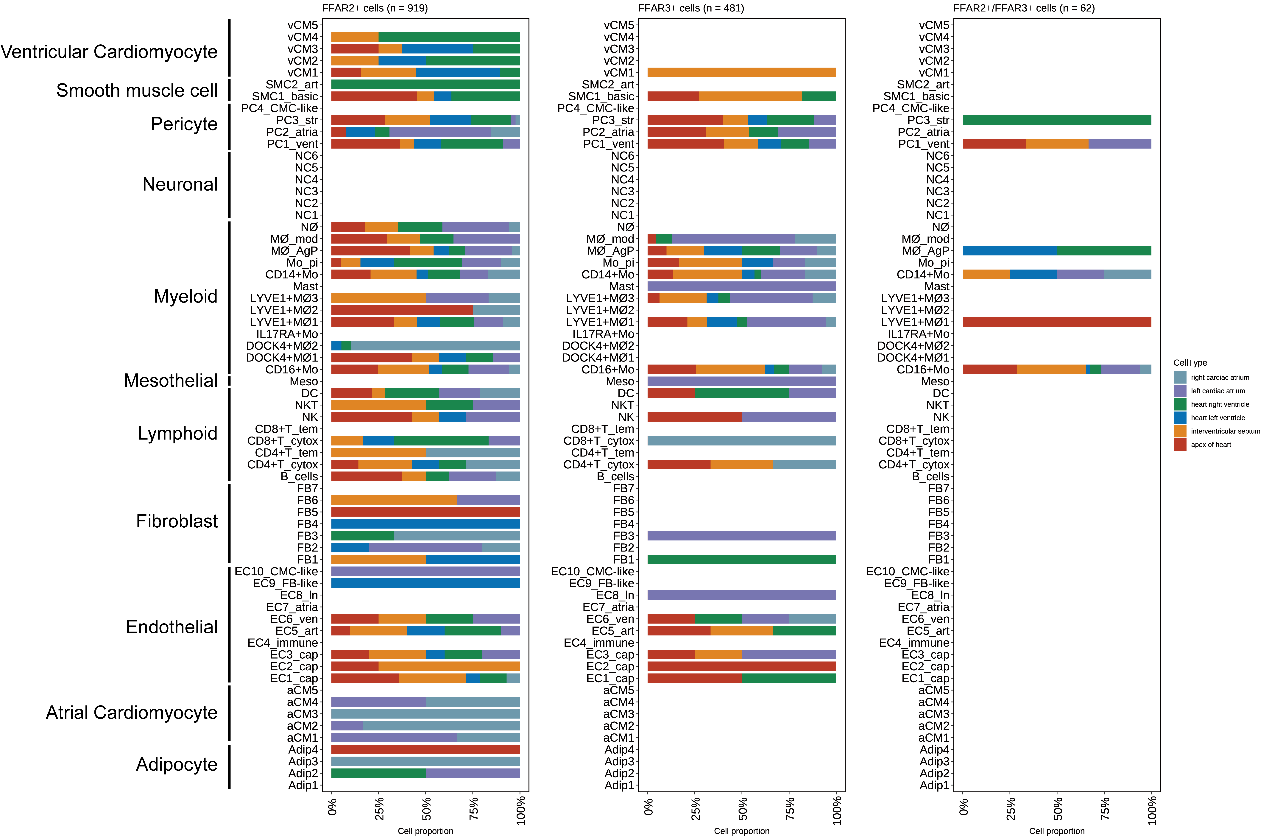
Supplemental Fig. 1 **| Subtype proportion of** FFAR2/3^+^ cells in the human heart.

The proportion of cells belonging to different heart regions for each subtype of FFAR2 positive cells (left panel), FFAR3 positive cells (middle panel), and double-positive cells (right panel) are shown.

## Supplemental Figure 2


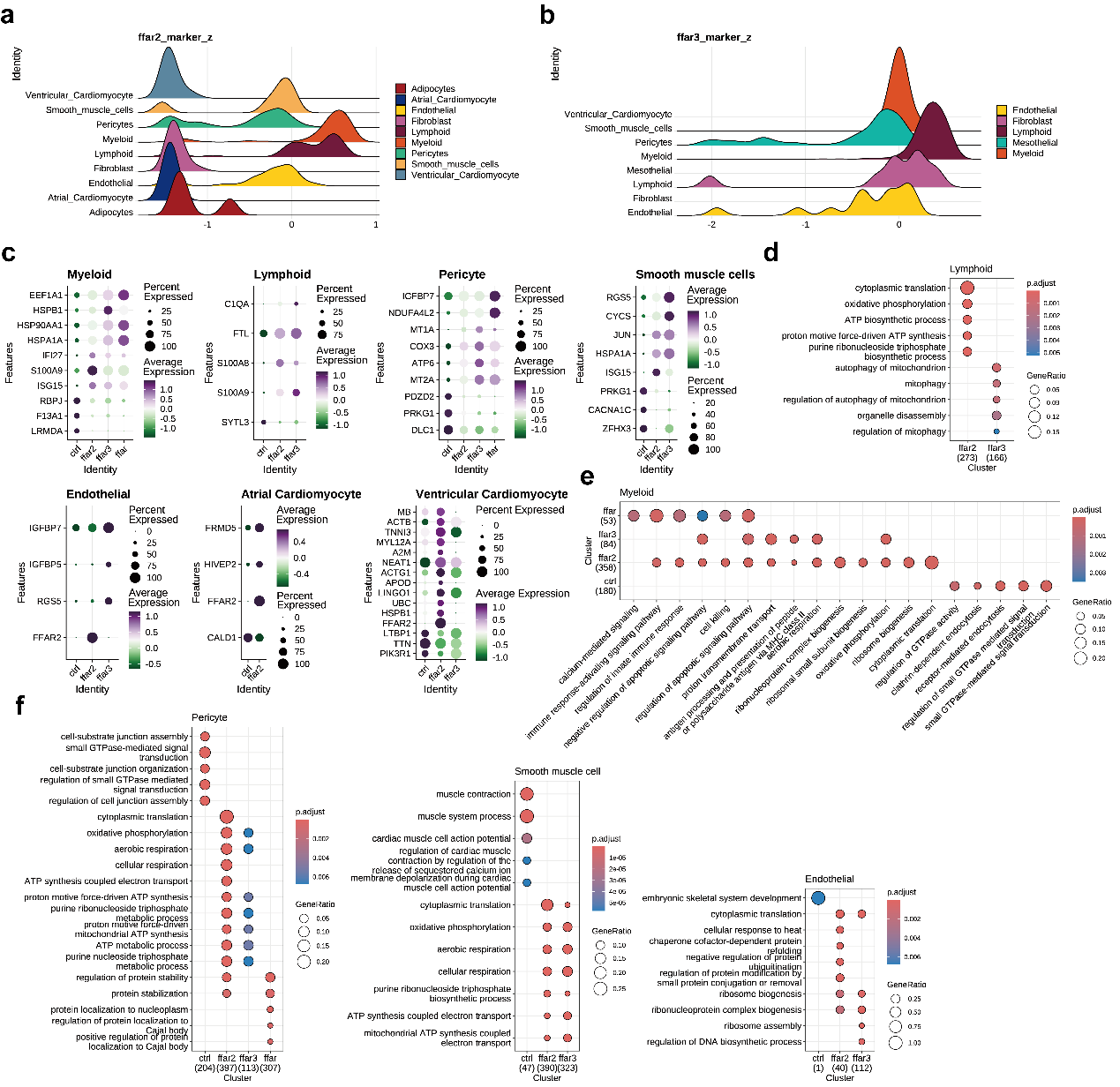
Supplemental Fig. 2 **| Gene expression pattern of** *FFAR2*/*3*^+^ cells in the human heart.

a-b, Gene set z-scores for *FFAR2*^+^ cells (a) and *FFAR3*^+^ cells (b) enriched genes plotted in the ridge plot. c, Dot plot of top marker genes in *FFAR2*^+^, *FFAR3*^+^, *FFAR2*^+^/*FFAR3*^+^, and *FFAR2*^-^/*FFAR3*^-^ cells for myeloid, lymphoid, pericyte, smooth muscle cells, endothelial, atrial cardiomyocytes, and ventricular cardiomyocytes. ctrl, *FFAR2*^-^/*FFAR3*^-^ cells; ffar2: *FFAR2*^+^ cells; ffar3: *FFAR3*^+^ cells; ffar: *FFAR2*^+^/*FFAR3*^+^ cells. The marker genes were identified with Wilcoxon tests as implemented in Seurat’s FindAllMarkers function. d, Dot plot of top enriched gene ontology biological process items for *FFAR2*^+^, *FFAR3*^+^, *FFAR2*^+^/*FFAR3*^+^, and *FFAR2*^-^/*FFAR3*^-^ lymphoid cells. e, Dot plot of top enriched gene ontology biological process items for *FFAR2*^+^, *FFAR3*^+^, *FFAR2*^+^/*FFAR3*^+^, and *FFAR2*^-^/*FFAR3*^-^ myeloid cells. f, Dot plot of top enriched gene ontology biological process items for *FFAR2*^+^, *FFAR3*^+^, *FFAR2*^+^/*FFAR3*^+^, and *FFAR2*^-^/*FFAR3*^-^ pericyte, smooth muscle cells, and endothelial cells.

## Supplemental Figure 3


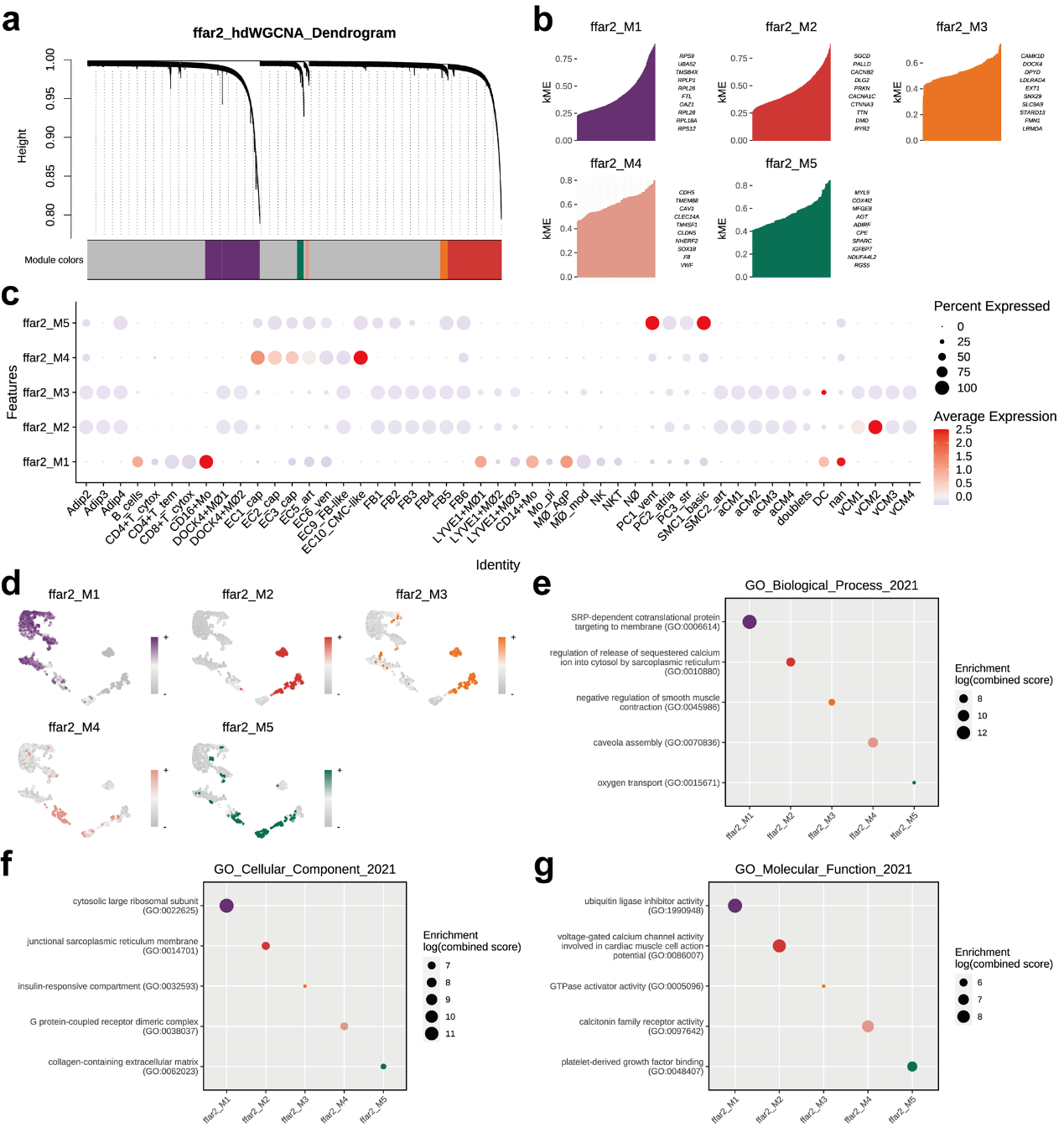
Supplemental Fig. 3 **| Gene expression modules of** *FFAR2*^+^ cells in the human heart.

**a, Co-expression network gene modules of *FFAR2*^+^ human heart cells calculated by the hdWGCNA R package and plotted in a dendrogram plot. Each leaf on the dendrogram represents a single gene, and the color at the bottom indicates the co-expression module assignment. b, The genes in each module are ranked by eigengene-based connectivity (kME). c, Dot plot showing the expression of gene modules in each cell type subcluster. d, UMAP embedding of gene modules in *FFAR2*^+^ human heart cells. e, Dot plot presented the top Gene Ontology (GO) biological process terms of each module. f, Dot plot presented the top cellular component GO terms of each module. g, Dotplot presented with the top molecular function GO terms of each module.**

## Supplemental Figure 4


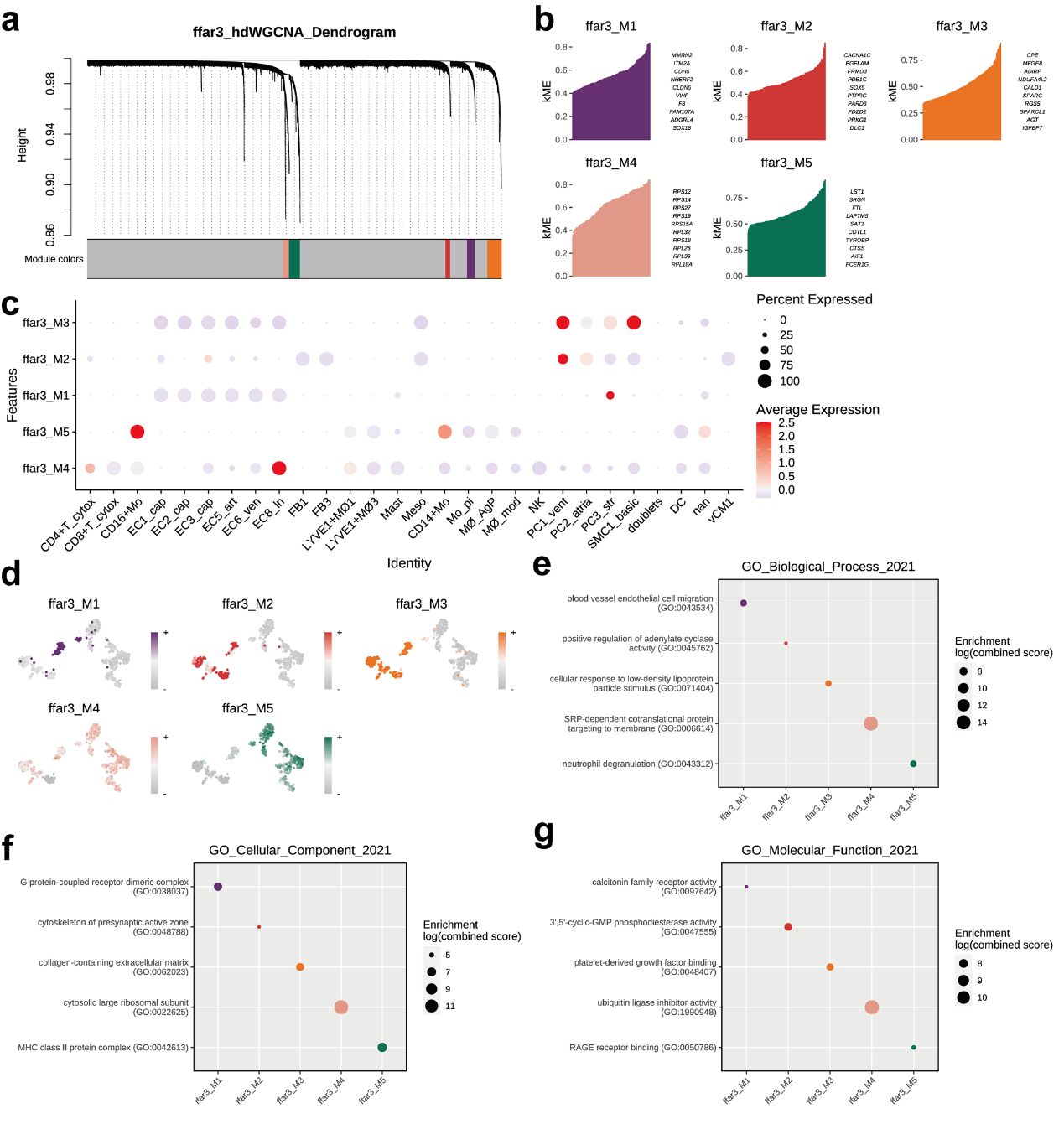
Supplemental Fig. 4 **| Gene expression modules of** *FFAR3*^+^ cells in the human heart.

**a, Co-expression network gene modules of *FFAR3***^+^ **human heart cells calculated by the hdWGCNA R package and plotted in a dendrogram plot. Each leaf on the dendrogram represents a single gene, and the color at the bottom indicates the co-expression module assignment. b, The genes in each module are ranked by eigengene-based connectivity (kME). c, Dot plot showing the expression of gene modules in each cell type subcluster. d, UMAP embedding of gene modules in *FFAR3***^+^ **human heart cells. e, Dot plot presented the top Gene Ontology (GO) biological process terms of each module. f, Dot plot presented with the top cellular component GO terms of each module.** **g, Dot plot presented with the top molecular function GO terms of each module.**

## Supplemental Figure 5

Supplemental Fig. 5 **| Immunofluorescent staining of** *FFAR2* and FFAR3 in human infarcted myocardium.

a, co-staining of *FFAR2* and *FFAR3* with CD68; b, co-staining of *FFAR2* with CD14 and CX3CR1; c, co-staining of *FFAR3* with CD14 and CX3CR1and CD68. Nuclei are counterstained with DAPI (dark blue). Scale bar, 5 μm.

## Supplemental Figure 6


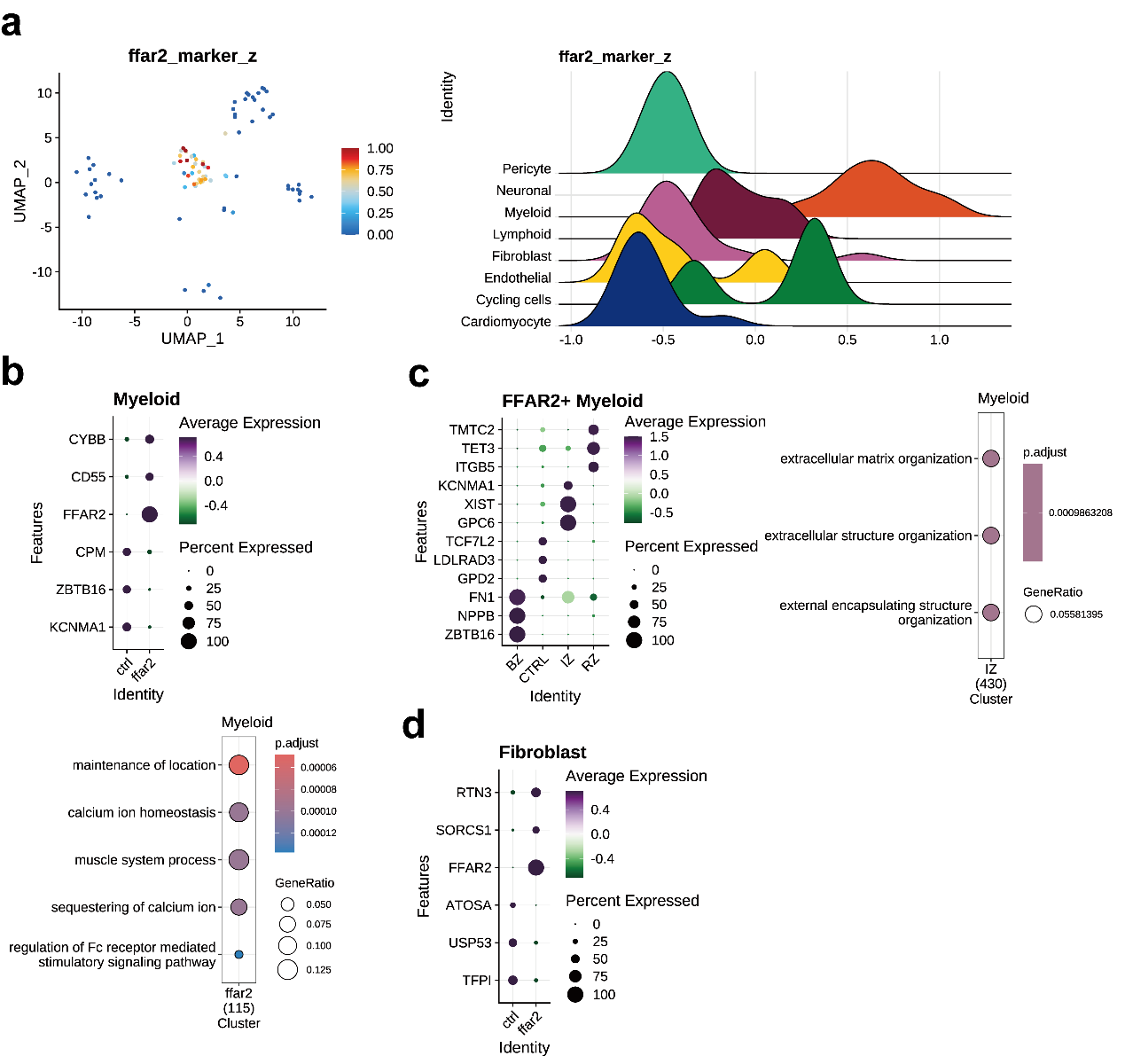
Supplemental Fig. 6 **| Gene expression pattern of** *FFAR2*^+^ cells in human myocardial infarction heart.

a, Gene set z-scores for *FFAR2*^+^ cells enriched genes plotted in the UMAP embedding (left panel) and ridge plot (right panel). b, The Dot plot of top marker genes (upper panel) and top enriched gene ontology biological processes (lower panel) in *FFAR2*^+^ (ffar2) and *FFAR2*^-^ (ctrl) myeloid cells. c, The Dot plot of top marker genes (left panel) and top enriched gene ontology biological processes (right panel) in different infarction-related cardiac regions of *FFAR2*^+^ myeloid cells. e, The top marker genes of *FFAR2*^+^ (ffar2) and *FFAR2*^-^ (ctrl) cells in fibroblast (left panel) and cycling cells (right panel). RZ, remote zone, the unaffected left ventricular myocardium; BZ, border zone; IZ, ischemic zone; FZ, fibrotic zone, human heart specimens at later stages after myocardial infarction; CTRL, control samples from non-transplanted donor hearts. The marker genes were identified with Wilcoxon tests as implemented in Seurat’s FindAllMarkers function.

## Supplemental Figure 7


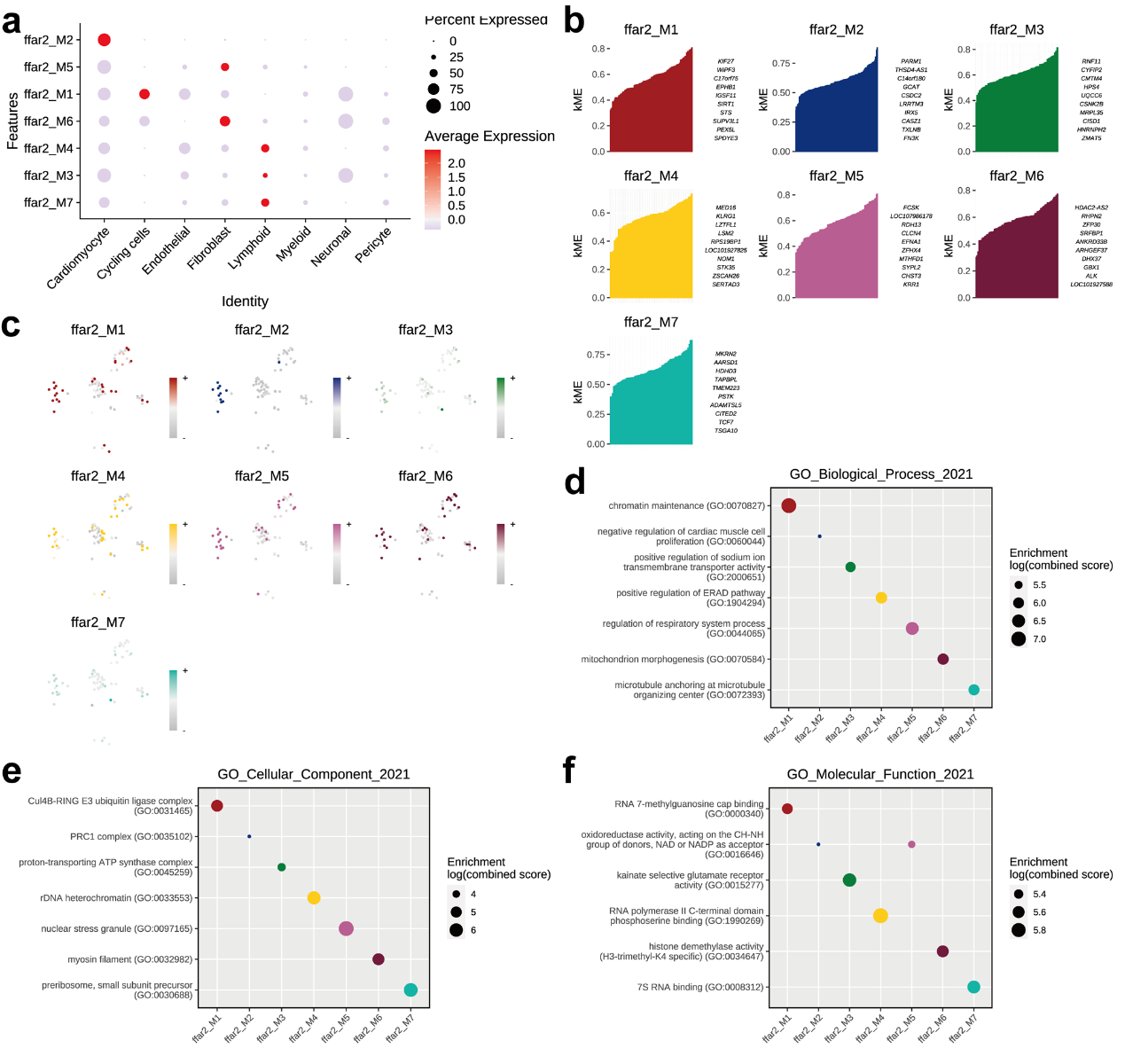
Supplemental Fig. 7 **| Gene expression modules of** *FFAR2*^+^ cells in human myocardial infarction heart.

**a, Dot plot showing the expression of gene modules in each cell type. b, The genes in each module are ranked by eigengene-based connectivity (kME). c, UMAP embedding of gene modules in *FFAR2*^+^ human heart cells. d, Dot plot presented with the top Gene Ontology (GO) biological process terms of each module. e, Dot plot presented the top cellular component GO terms for each module. f, Dot plot presented with the top molecular function GO terms of each module.**

## Supplemental Figure 8


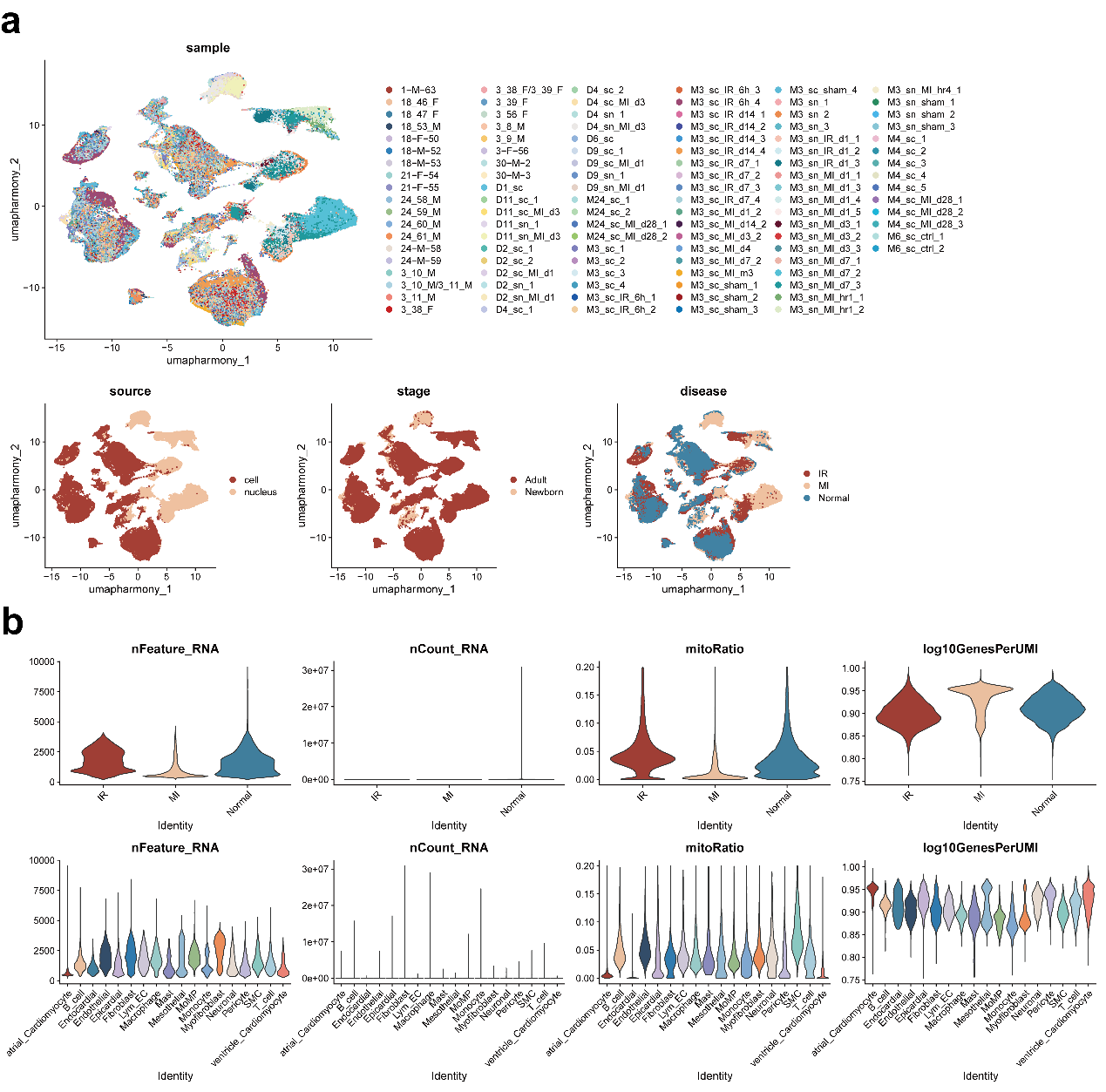
Supplemental Fig. 8 **| Characteristic of the integrated mice heart dataset.**

a, The UMAP embedding of mice heart cells divided by samples, cell sources, development stage, and diseases. There were 15 datasets integrated into one cell atlas, consisting of 283,704 cells. b, The nCount_RNA, nFeature_RNA, mitoRatio, and log10GenesPerUMI split by disease group and cell type.

## Supplemental Figure 9


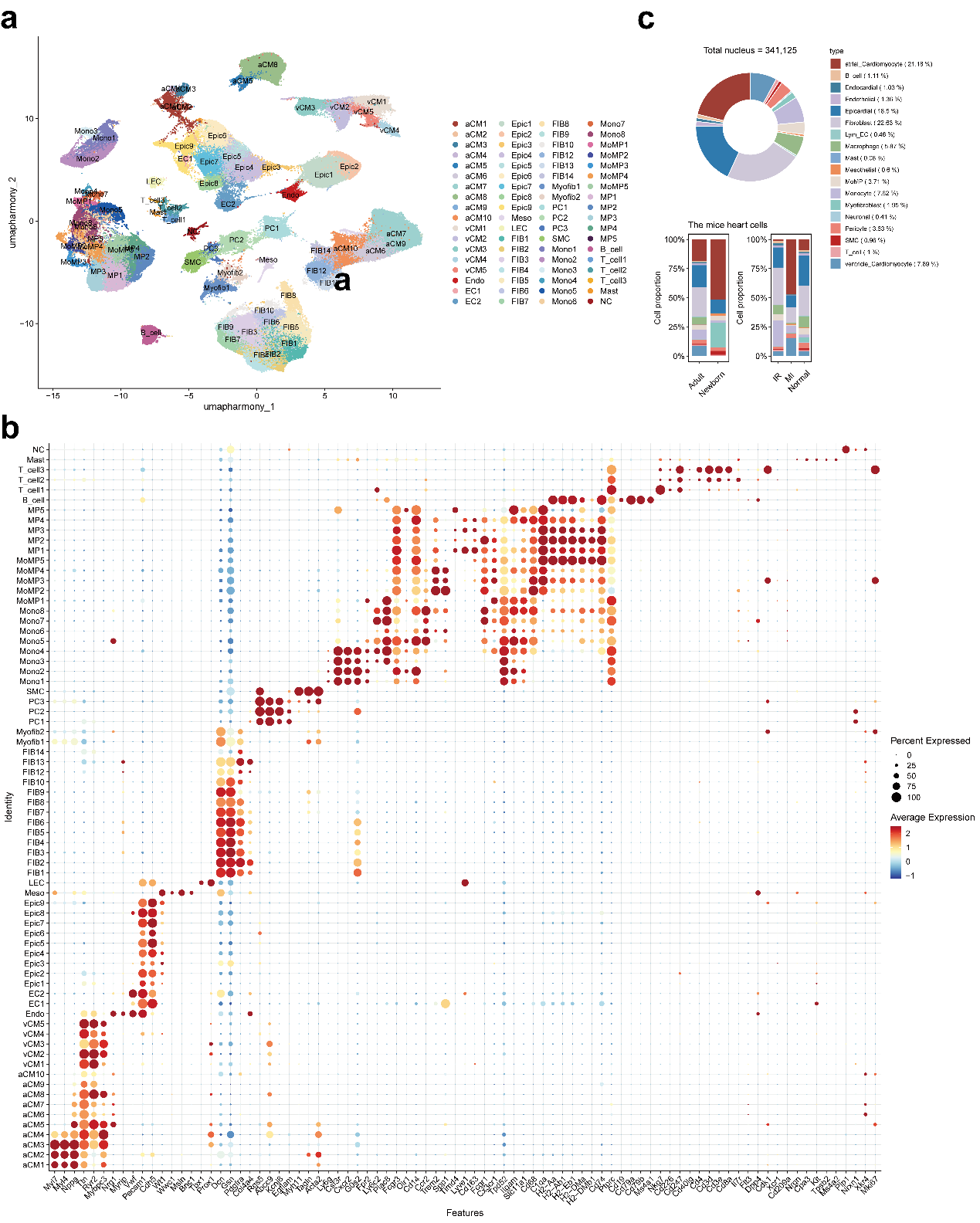
Supplemental Fig. 9 **| Cell types and subtypes of the integrated mice heart dataset.**

**a,** The UMAP embedding of mice heart cells divided by cell subtype. **b, Dot plot showing the expression of canonical marker genes in each** cell subtype. **c, The circle plot presented the proportion of cell types in mice heart cells, and the bar plot presented the proportion of cell types in either ischemic-reperfusion injury (IR), myocardial infarction (MI), and normal hearts.**

## Supplemental Figure 10


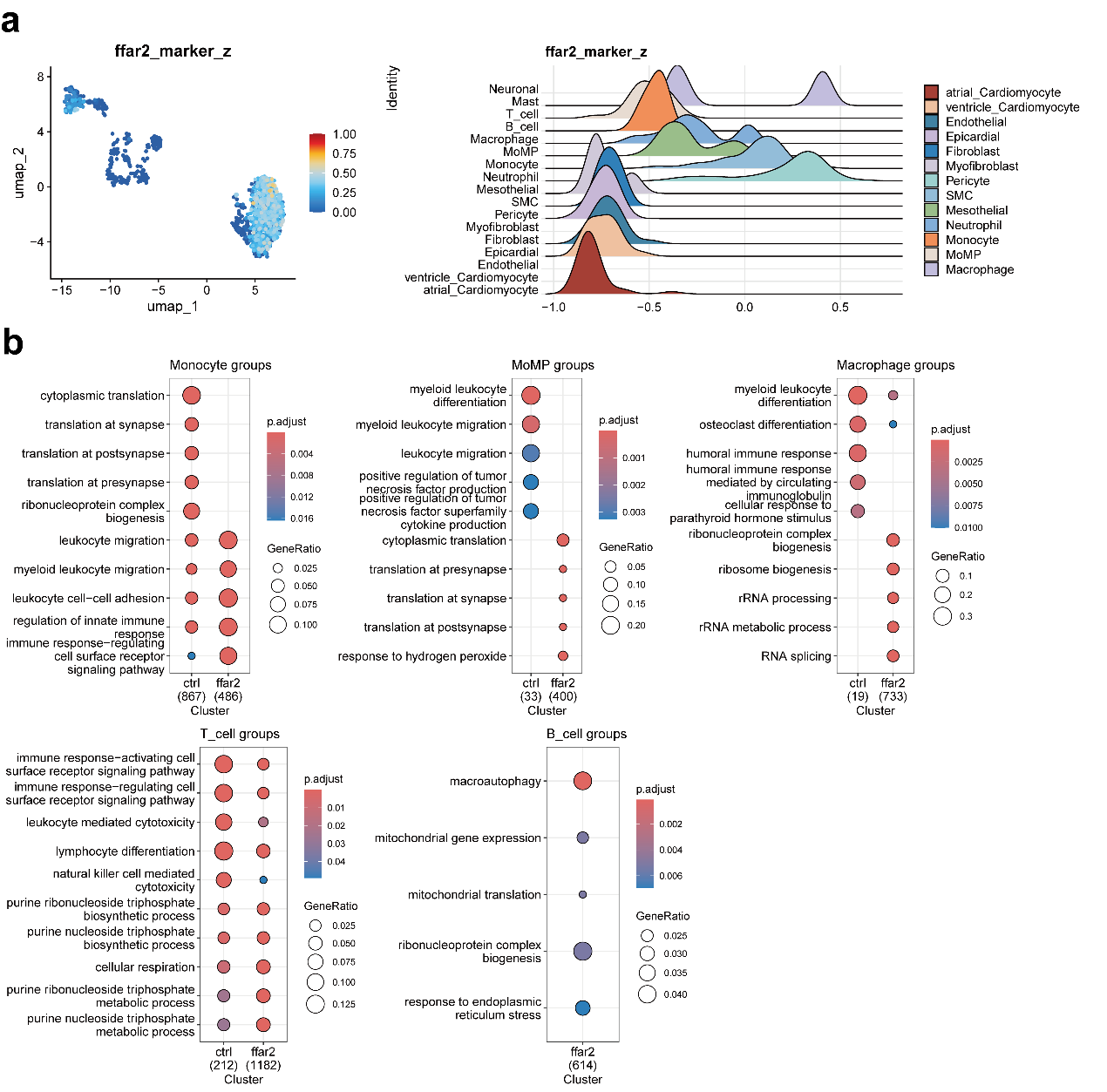
Supplemental Fig. 10 **| Gene expression pattern of** FFAR2^+^ cells in **mice** heart.

a, Gene set z-scores for *FFAR2*^+^ cells enriched genes plotted in the UMAP embedding (left panel) and ridge plot (right panel). b, The Dot plot of top enriched gene ontology biological processes in *FFAR2*^+^ (ffar2) and *FFAR2*^-^ (ctrl) group of macrophages, monocytes, dendritic cells, T cells, cycling cells, and cardiomyocytes.

## Supplemental Figure 11


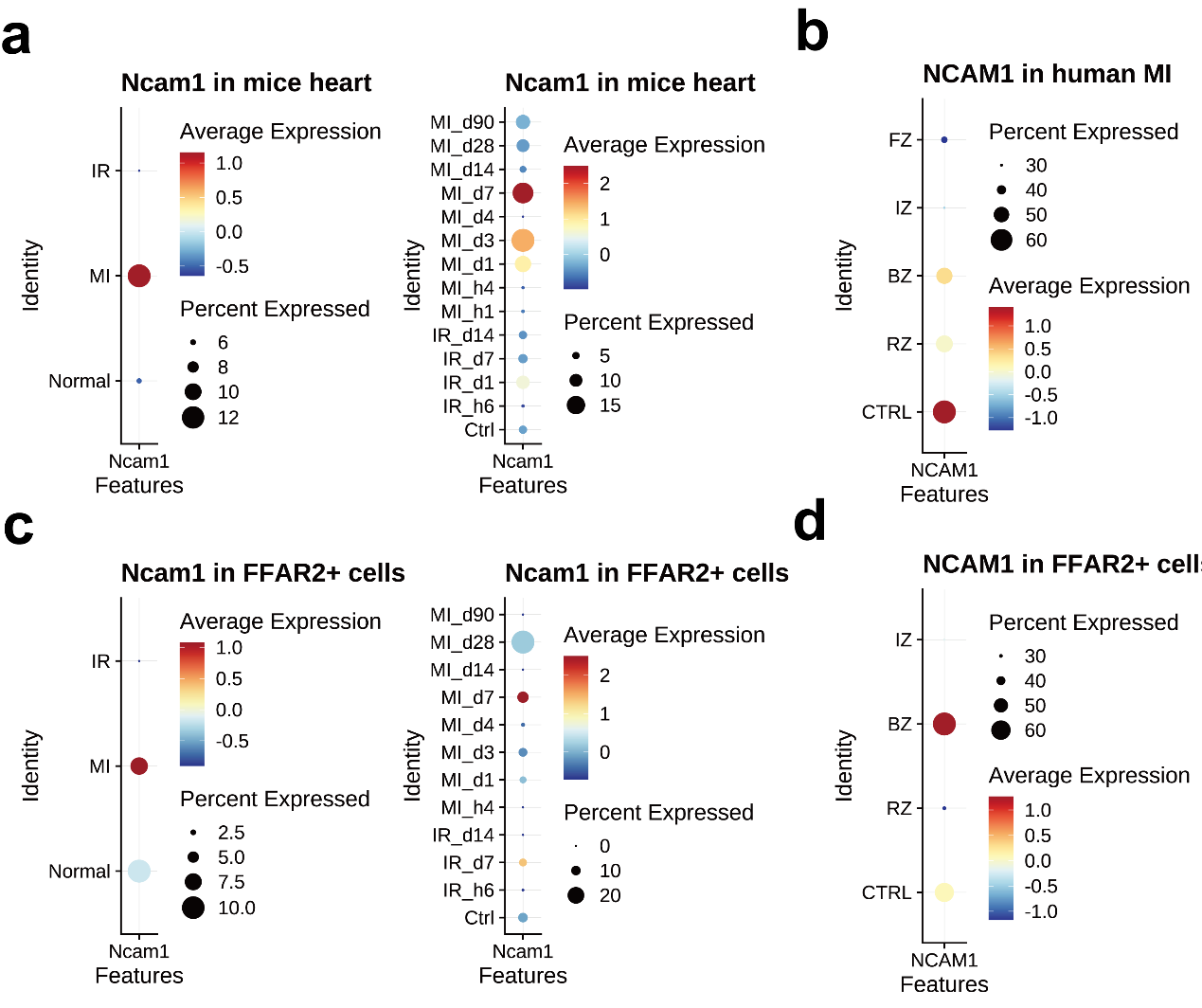
Supplemental Fig. 11 **| Gene expression pattern of** FFAR4 in mice heart.

**a-b, Dot plot showing the expression of Ncam1 in mice hearts (a) and human myocardial infarction hearts (b). c-d, Dot plot showing the expression of Ncam1 in** *FFAR2*^+^ cells **of mice hearts (c) and human myocardial infarction hearts (d).**
